# Polyphenolic Composition, Antioxidant, Antiproliferative and Antidiabetic Activities of *Coronopus didymus* Leaf Extracts

**DOI:** 10.3390/molecules27196263

**Published:** 2022-09-23

**Authors:** Saima Muzammil, Yunsheng Wang, Muhammad Hussnain Siddique, Errum Zubair, Sumreen Hayat, Muhammad Zubair, Arpita Roy, Rabia Mumtaz, Muhammad Azeem, Talha Bin Emran, Muhammad Qasim Shahid

**Affiliations:** 1 Department of Microbiology, Government College University, Faisalabad 38000, Pakistan; 2 School of Health and Life Science, Kaili University, Kaili 566011, China; 3 Department of Bioinformatics and Biotechnology, Government College University, Faisalabad 38000, Pakistan; 4 Department of Biotechnology, School of Engineering & Technology, Sharda University, Greater Noida 201310, India; 5 Department of Pharmacy, BGC Trust University Bangladesh, Chittagong 4381, Bangladesh; 6 Department of Pharmacy, Faculty of Allied Health Sciences, Daffodil International University, Dhaka 1207, Bangladesh; 7 College of Agriculture, South China Agricultural University, Guangzhou 510642, China

**Keywords:** natural medicine, MTT assay, DPPH assay, α-glucosidase assay, HepG_2_

## Abstract

*Coronopus didymus* (Brassicaceae) commonly known as lesser swine cress has been reported to be used for its pharmacological activities. This study aimed to evaluate the medicinal potential of *C. didymus* extracts against cancer, diabetes, infectious bacteria and oxidative stress and the identification of bioactive compounds present in these extracts. The effects of using different solvents for the extraction of *C. didymus* on the contents of major polyphenols and biological activities were investigated. Plant sample was shade dried, ground to a fine powder, and then soaked in pure acetone, ethanol and methanol. The highest contents of major polyphenols were found in methanol-based extract, i.e., chlorogenic acid, HB acid, kaempferol, ferulic acid, quercetin and benzoic acid with 305.02, 12.42, 11.5, 23.33, 975.7 and 428 mg/g of dry weight, respectively, followed by ethanol- and acetone-based extracts. The methanol-based extract also resulted in the highest antioxidant activities (56.76%), whereas the highest antiproliferative (76.36) and alpha glucosidase inhabitation (96.65) were demonstrated in ethanol-based extracts. No antibacterial property of *C. didymus* was observed against all the tested strains of bacteria. Further studies should be focused on the identification of specific bioactive compounds responsible for pharmacological activities.

## 1. Introduction

History of plant-based medicine is as old as human history. Evidence of such practices dates to prehistoric times. Clay tablets were found from the period of the Sumer civilization 1700 BC with a record of 12 drug recipes of more than 250 plants. Egyptians in 1550 BC documented their knowledge regarding 800 prescriptions from 700 plants in the Ebers Papyrus [1]. Knowledge of medicinal plants is based on hundreds, if not thousands of years of empirical research. Every civilization in history has adapted their own method of addressing various diseases depending upon their faith, custom, culture and experience [2]. Some of these practices were found in common throughout history. This may be due to the fact that traditional knowledge and experience of folk medicine is preserved in the form of writings and drawings in caves, monuments, paper or any written source available from that time. Moreover, it is inherited from generations to generations and has grown over the years through personal observation and trial and error method. However, due to the emergence of new medicinal practices and drugs, most of the knowledge of indigenous medicine has been lost or is at the risk of being lost [3].

In the mid-20th century, traditional medicine was phased out from mainstream medicine because it became less profitable than newly synthetic drugs [4]. The promotion of plant-based medicine by the World Health Organization in developing countries to meet the requirements not met by modern system has increased the use of traditional medicine globally [5]. A growing concern for the side effects of modern medicine is increasing the number of people seeking refuge in conventional medicine, making it more valuable economically and commercially. According to the World Health Organization, 80% of the world population of developing countries rely completely on conventional medicine for their basic health needs. Belief in safety, cost effectivity and easy accessibility to plant-based medicine is globally boosting this industry with an annual growth rate of 15% [6].

There has been an increasing interest in the identification of biochemicals from plants and the study of the pharmacological potential of plant extracts and bioactive compounds in the past few years [7,8,9]. Polyphenols have been reported to be the major bioactive compounds in plants used to treat diabetes, cancer and oxidative stress.

*Coronopus didymus* a weed of the Brassicaceae family is commonly known as “wartcress” or “swinecress” [10]. The plant has traditionally been used to treat cough, bruises, traumas, arthrosis, bronchitis, external ulcers, cancer, rheumatism, gastric and urinary problems. It has been used to relieve muscle pain, minimize fever and inflammation, purify blood and clear catarrh [11,12]. *Coronopus didymus* has also been reported to have antifungal, antioxidant, antimalarial, antitumor, wound healing and anti-inflammatory properties [13,14]. Apart from therapeutic and curative properties, because of its high biomass it is reported to have phytoremediation property for zinc [15], lead [16] and cadmium [16].

*Coronopus didymus* is an abundant source of bioactive compounds which have been reported to possess inhibitory activities against cancer and the alpha glucosidase enzyme [17,18]. These bioactive compounds are very sensitive to external conditions provided at the time of growth. Moreover, extraction conditions majorly using solvent and drying temperature also affect the amount and nature of bioactive compounds and their derivatives [19,20].

Diabetes mellitus is a group of diseases characterized by a high blood glucose level due to defects in the action and secretion of insulin. Chronic hyperglycemia results in the failure, damage and dysfunction of various body organs. It mostly affects the heart, eyes, kidneys, blood vessels and nerves. International Diabetes Federation’s (IDF) statistics indicate that 1 in every 11 has diabetes in the world. The prevalence of diabetes mellitus is from 7.66% to 11%. It has been projected that this would increase to 15% in 2030. Diabetes is a progressive disease which wears out body functions gradually and vital body parts [21].

Cancer is also a continual battle around the world. Cancer occurs when cellular changes throughout the body cause unrestricted growth and cell division. According to a report in 2015 by World Health Organization (WHO), cancer is the first or second major cause of death before 70 years in 91 out of 172 countries and occupies a third or fourth place in 22 additional countries [22].

Almost all the life processes and functions, from metabolism to bioenergetics involve oxidation–reduction reactions. Therefore, it can be said that equilibrium in redox reactions is vital for life. Biological systems are under a constant attack of reactive oxygen species. To defend such attacks, the body has a complex mechanism of regulating antioxidants, but when this system collapses, the biological system has to face oxidative stress which may lead to several diseased conditions such as cancer, aging, diabetes, inflammatory joint diseases and heart diseases [23,24]. This study aims to evaluate the medicinal potential of *C. didymus* extracts against cancer, diabetes, infectious bacteria and oxidative stress as well as the identification of major bioactive compounds present in these extracts.

## 2. Results

### 2.1. Phytochemical Analysis

High-performance liquid chromatography (HPLC) was performed for the identification and quantification of polyphenols (Table 1). Among the nine major identified polyphenols, chlorogenic acid, benzoic acid and quercetin were present in all the extracts with varying concentrations. Chlorogenic acid at 2.84 min, kaempferol at 11.02 min, ferulic acid at 12.49 min, coumarin at 16.74 min and benzoic acid at 18.17 min were identified in acetone-based extracts. Chlorogenic acid at 2.98 min, HB acid at 6.93 min, caffeic acid at 7.24 min, benzoic acid at 18.78 min and rutin at 23.89 min were identified in ethanol-based extracts. Chlorogenic acid at 2.7 min, HB acid at 6.64 min, kaempferol at 11.13 min, ferulic acid at 12.64 min, quercetin at 16.91 min and benzoic acid at 18.38 min were identified in methanol-based extracts.

### 2.2. Antioxidant Activity

**Figure 1:**
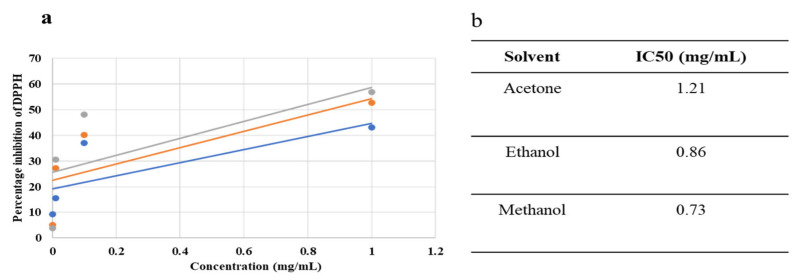
Dose–response curve (**a**) and IC_50_ values (**b**) of acetone-, ethanol-, and methanol-based extracts.

Different concentrations (1 mg/mL, 0.1 mg/mL, 0.01 mg/mL, 0.001 mg/mL) of plant extracted in three solvents (acetone, ethanol, methanol) were evaluated for their free radical scavenging ability using 0.3 mM DPPH. Gallic acid (0.3 mM) was used as a standard. All the tested concentrations exhibited significant radical scavenging activity in a concentration-dependent manner as shown in Table 2. The highest concentration of 1 mg/mL of methanol- and ethanol-based extracts demonstrated a maximum radical scavenging activity of 56.7% and 52.7%. The lowest concentration of 0.01 mg/mL exhibited almost negligible antioxidant activity. Extracts dissolved in methanol and ethanol performed better than the extracts dissolved in acetone with IC_50_ values of 0.73 mg/mL and 0.86 mg/mL, respectively (Figure 1).

### 2.3. Antidiabetic Activity

**Figure 2:**
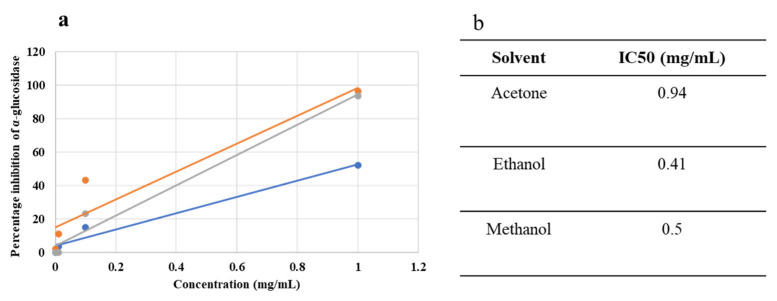
Dose–response curve (**a**) and IC_50_ values (**b**) of acetone-, ethanol-, and methanol-based extracts.

The antidiabetic activity of *C. didymus* was evaluated using the alpha glucosidase enzyme, one of the key enzymes involved in Type II diabetes. Acarbose was used as a standard. All the extracts demonstrated a significant antidiabetic activity in a concentration-dependent manner as shown in Table 3. The highest activity was exhibited by the highest concentration (1 mg/mL) of all the extracts prepared in (acetone, ethanol, methanol) whereas no or an almost negligible activity was recorded by the lowest concentration (0.001 mg/mL) of all the extracts. The best alpha glucosidase inhibition activity was demonstrated by 1 mg/mL of ethanol extract (IC_50_ 0.41) followed by methanol extract (IC_50_ 0.50) (Figure 2).

### 2.4. Antiproliferative Activity

**Figure 3:**
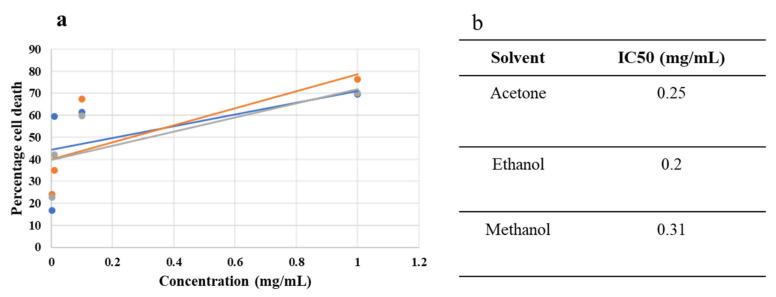
Dose–response curve (**a**) and IC_50_ values (**b**) of acetone-, ethanol-, and methanol-based extracts.

**Figure 4:**
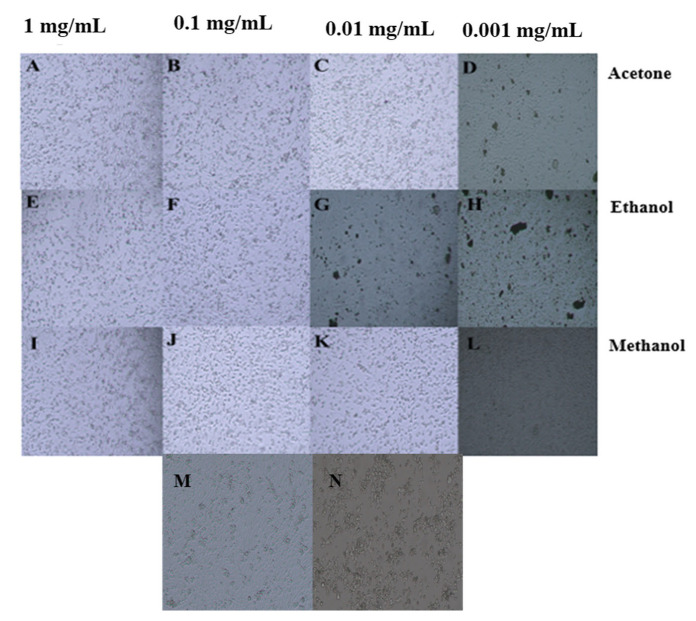
Antiproliferative activity of *Coronopus didymus* against HepG2 cells. (**A**–**D**) represent treatment with acetone-based extracts at a concentration of 1 mg/mL, 0.1 mg/mL, 0.01 mg/mL and 0.001 mg/mL, respectively. (**E**–**H**) depict treatment with ethanol-based extracts at a concentration of 1 mg/mL, 0.1 mg/mL, 0.01 mg/mL and 0.001 mg/mL, respectively. (**I**–**L**) are treatment with methanol-based extracts at a concentration of 1 mg/mL, 0.1 mg/mL, 0.01 mg/mL and 0.001 mg/mL, respectively. (**M**,**N**) represent negative and positive control, respectively.

The antiproliferative potential of *C. didymus* extracts was evaluated using an MTT assay. For this purpose, different concentrations (1 mg/mL, 0.1 mg/mL, 0.01 mg/mL, 0.001 mg/mL) of plant extracted in acetone, ethanol and methanol were exposed to the HepG2 cancer cell line. All the concentrations exhibited potential antiproliferative activity against HepG2 cells in a concentration-dependent manner as shown in Table 4. The IC_50_ values were 0.20 mg/mL for ethanol, 0.31 mg/mL for methanol and 0.25 mg/mL for acetone (Figure 3). The maximum activity was recorded by 1 mg/mL of extract prepared in ethanol (76.36%). The extracts prepared in methanol and acetone at a concentration of 1 mg/mL inhibited cell proliferation by 70.22% and 69.59%, respectively. The effect on morphology of HepG2 cells are shown in Figure 4.

### 2.5. Antibacterial Activity

As reported by [25,26], *C. didymus* was found to have no antibacterial property against *Escherichia coli, Klebsiella pneumoniae* and *Staphylococcus aureus*. Previous studies did not include work on *Acinetobacter*, and we found that *Acinetobacter* was also resistant to *C. didymus*.

## 3. Discussion

In the past few decades, medicinal plants have often been investigated for a potential anticancer effect by an evaluation of their ability to counteract cancer cells in vitro. For the first time, we reported the effects of the solvent used for the extraction on antiproliferative, antioxidant and antidiabetic activities as well as on the contents of major polyphenols from *C. didymus*. In the present study, extracts of *C. didymus* significantly decreased the proliferation of the HepG2 cell line, thus confirming previous reports of cytotoxic properties [27]. The antiproliferative activity of *C. didymus* against HeLa and LN18 cell lines have been attributed to the presence of polyphenols. Oxidative reduction is a potential phenomenon that allows phenolic compounds to have antiproliferative effects, carcinogen inactivation and metastasis inhibition [27,28].

Free radicals interact with other molecules and initiate larger chemical reactions in the human body, which leads to various diseased conditions. It was found that the antioxidant potential of the tested extracts increased with the increasing polarity potential of the solvents used for the extraction, similar to the results reported in a study by [13]. The antioxidant activity can be attributed to the presence of polyphenols in *C. didymus*. As most of the phenolic compounds are polar in nature, they can be extracted with high yield in a higher polarity solvent such as methanol and ethanol.

An important role of plant extracts as potent inhibitor of α-glucosidase has been suggested in previous studies investigating antidiabetic properties [29]. Acarbose is widely used for α-glucosidase but has been reported to have hepatotoxic and nephrotoxic side effects. The percentage inhibition value of ethanol extract of *C. didymus* (1 mg/mL) was even higher than that of acarbose used as a standard (59.32%). This shows that *C. didymus* can be an affective inhibitor for glucose that rises after a meal, as alpha glucosidase is the main enzyme involved in increasing the concentration of postprandial blood glucose levels. Furthermore, alpha glucosidase drug inhibitors have various side effects; one of them is getting absorbed in systemic circulation and regulating nonpathogenic protein molecules [30].

The biological properties of plants have been attributed to the presence of polyphenols and other bioactive compounds in different parts of the plants. Various studies have reported the effectiveness of polyphenols as an antioxidant, anticancer, anti-inflammatory and wound healing agents [19,31]. The contents of major bioactive compounds extracted from the plant are significantly affected by the solvents used, which suggests that the type of solvent might help to improve the concentration [19,20]. While comparing the contents of polyphenols using different solvents, the acetone-, ethanol- and methanol-based extracts have proven to be the most efficient alternatives, with acetone producing the highest yields for the extraction of several raw materials [32,33,34]. However, methanol- and ethanol-based extracts have proven more efficient in some other studies, e.g., for the extraction of polyphenols in *Hibiscus sabdariffa* [35]. The present study showed the highest contents of major polyphenols were found in methanol-based extracts followed by ethanol- and acetone-based extracts. The variation in contents of polyphenolic compounds using different solvents might be attributed to the difference of polarity of phenolic compounds. The addition of water to organic solvents such as acetone, methanol and ethanol creates a more polar medium, which sometimes increases the yield of phenolic compounds even further [36]. The healing properties of *C. didymus* can be attributed to the presence of different phytochemicals.

## 4. Materials and Methods

### 4.1. Phytochemical Analysis

The phytochemical analysis of *C. didymus* dissolved in pure ethanol, methanol and acetone was performed using high-performance liquid chromatography (HPLC). A 20 µL sample was injected in hydro-RP 80A (250 mm × 4.60 mm, 4 µm) at room temperature. A mixture of acetic acid (1%) and acetonitrile (5%) in water (solution A) and methanol (5%) in acetonitrile (solution B) were used as the mobile phase in gradient 82% A and 18% B. An isocratic elution was performed for 40 min at a flow rate of 1 mL/min. The quantification of the phenolic compounds from three solvents was performed at 280 nm. The analysis was performed under stable chromatographic conditions.

### 4.2. Preparation of Plant Extract

*Coronopus didymus* was collected from the campus of Government College University, Faisalabad. The plant sample was washed and shade dried. The whole plant sample was ground to a fine powder. The extraction was carried out by soaking 1 g of ground plant sample in pure acetone, ethanol and methanol. After overnight incubation in a shaking incubator, the slurry was filtered with a Whatman No. 1 filter paper. The solvent from the filtered solution was evaporated and the extract was dissolved in 1 mL of phosphate buffer saline to make a concentration of 1 mg/mL. Three more concentrations 0.1 mg/mL, 0.01 mg/mL and 0.001 mg/mL were made by further diluting 1 mg/mL in phosphate buffer saline (PBS). A total of four concentrations of plant sample were used for further studies. The extracts were stored at −20 °C until used for further analysis.

### 4.3. Antidiabetic Activity

Vongsak and Kongkiatpaiboon’s [3] experiment with modifications was performed to evaluate the inhibition activity of *C. didymus* against the alpha glucosidase enzyme. The plant extract (12.5 µL dissolved in PBS) and 40 µL of enzyme α-glucosidase (0.5 U/mL) with acarbose 10 mM (12.5 µL) as a standard were mixed in 140 µL of PBS (pH 7.4) for 5 min at 37 °C. The inhibition activity of the enzyme was analyzed by the breakdown of a 40 µL substrate of p-nitrophenyl-α-D-glucopyranoside (5 mM) for 30 min at 37 °C. A microplate reader was used to record the enzyme activity at an absorbance of 405 nm. The percentage inhibition was calculated using the formula:

(1) $$\left[\left(rate\;of\;measured\;normal\;activity-rate\;of\;measured\;inhibited\;activity\right)/\;rate\;of\;measured\;normal\;activity\;\times 100\right]$$

to find the inhibition activity of plant extracts for the enzyme alpha glucosidase.

### 4.4. Antiproliferative Activity

The antiproliferative activity of *C. didymus* extracts was evaluated using an MTT (3-(4,5-dimethyl-2-yl)-2,5-diphenyltetrazolium bromide) assay on HepG2 cells. A human liver cancer HepG2 cell line was grown as a monolayer in Dulbecco’s Modified Eagle Medium (DMEM) supplemented with 10% fetal bovine serum (FBS), 1% glutamine and 1% penicillin–streptomycin at 37 °C with 5% CO_2_ in a humified incubator. A cell density of 1 × 10^4^ cells/well was seeded in a 96-well plate. Confluent cells were treated with 3 µL of plant sample for 48 h. The cell proliferation activity was assessed by MTT dye (0.5 mg/mL). Purple-colored formazan crystals were dissolved in 150 µL of dimethyl sulfide (DMSO) for 4 h at 37 °C. Doxorubicin (10 µM) was used as a positive control and PBS was used as a negative control. A microplate enzyme-linked immunosorbent assay (ELISA) reader was used to measure the optical density at 630 nm. The antiproliferative activity was evaluated by calculating the percentage inhibition of plant extracts inhibiting the growth of human liver cancer HepG2 cell line. Pictures of the cells after treatment were taken by an inverted microscope using 40× magnification.

### 4.5. Antioxidant Activity

The free radical scavenging activity of *C. didymus* extracts was assessed by a 2,2-Diphenylpicrylhydrazyl (DPPH) assay as reported by [37] with some modifications. A plant extract of 10 µL was treated with 190 µL of DPPH (0.3 mM). Gallic acid (0.3 mM) was used as a standard. After 30 min, the absorbance was recorded at 490 nm due to the nonavailability of a 517 nm filter of the microplate reader. The free radical scavenging ability of plant extracts was calculated by the formula of percentage inhibition.

### 4.6. Antibacterial Activity

Antibacterial activity of *C. didymus* extracts was determined using the well diffusion method on Mueller–Hinton agar (MHA) [38]. Stock cultures of Escherichia coli, Acinetobacter, Klebsiella pneumoniae and Staphylococcus aureus were grown in nutrient broth for 24 h at 37 °C from single colonies appearing on an agar plate. A plant extract of 15 µL was inoculated in bacterial swabbed wells (6 mm) on MHA plates in an aseptic environment. The diameter of growth inhibition zone was measured after 24 h of incubation at 37 °C.

### 4.7. Statistical Analysis

Each experiment was performed in triplicate and data are presented as mean *±* standard deviation. The statistical analysis was performed using graphpad prism software. An analysis of variance and Tukey’s post hoc test were performed for the comparison among different treatment groups.

## 5. Conclusions

The present study suggested that the polyphenolic compounds in the *C. didymus* might be the major contributor to the latter’s antioxidant, α-glucosidase inhibition and to its antiproliferative activity since the efficiency of the tested extracts was closely reflected by the varying contents of these compounds. However, there might be other compounds, e.g., nonpolar compounds (soluble in ethanol, methanol and acetone), contributing to the pharmacological activities of *C. didymus*. Overall, the extraction with methanol yielded the highest concentrations of the identified polyphenols. This study paves the way for further in vivo and clinical trials. Further studies are, however, needed to identify the specific compounds responsible for the biological activity of *C. didymus*. The study also demonstrated that above-mentioned bacteria were resistant to *C. didymus* extracts.

## Figures and Tables

**Table 1:** Phenolic compounds identified in *Coronopus didymus* leaf extracts.

Compound	Retention Time (min)	Concentration (ug/g Mean **±** **Standard** Deviation)		
		Acetone-Based Extract	Ethanol-Based Extract	Methanol-Based Extract
Chlorogenic acid	2.88	42.71 ± 1.67	56.48 ± 2.28	305.02 ± 6.34
kaempferol	11.07	2.56 ± 0.54	ND	11.50 ± 1.74
Ferulic acid	12.46	5.59 ± 0.89	ND	23.33 ± 1.06
Coumarin	16.085	88.7 ± 3.90	ND	ND
Benzoic acid	18.30	50.65 ± 2.51	330.23 ± 6.50	428.7 ± 6.61
HB acid	6.75	ND	16.39 ± 1.82	12.42 ± 1.04
Caffeic acid	7.49	ND	3.80 ± 0.21	ND
Rutin	23.89	9.43 ± 1.23	10.61 ± 1.92	ND
Quercetin	16.91	271.5 ± 4.92	432.1 ± 7.09	975.7 ± 7.63

**Table 2:** Free radical scavenging activity of *Coronopus didymus* using DPPH assay (mean ± standard deviation).

Solvent	1 mg/mL	0.1 mg/mL	0.01 mg/mL	0.001 mg/mL
Acetone	43.06 ± 1.15	36.96 ± 1.13	15.50 ± 1.43	9.29 ± 1.47
Ethanol	52.73 ± 1.73	40.10 ± 1.50	27.12 ± 1.58	5.04 ± 0.57
Methanol	56.76 ± 0.57	48.13 ± 0.51	30.60 ± 0.52	3.87 ± 0.58
Gallic acid (0.3 mM)	69.20 ± 1.40			

**Table 3:** Inhibitory effect of *Coronopus didymus* extracts (mean ± standard deviation) against alpha glucosidase.

Solvent	1 mg/mL	0.1 mg/mL	0.01 mg/mL	0.001 mg/mL
Acetone	52.15 ± 2.89	14.77 ± 1.44	3.38 ± 2.82	0.03 ± 0.55
Ethanol	96.65 ± 1.67	43.15 ± 1.94	10.99 ± 3.17	2.06 ± 1.78
Methanol	93.58 ± 1.27	23.09 ± 0.62	0.1 ± 1.30	0.09 ± 0.32
Acarbose (10 mM)	59.39 ± 1.47			

**Table 4:** Antiproliferative activity of *Coronopus didymus* extracts (mean ± standard deviation) on HepG2 cell line.

Solvent	1 mg/mL	0.1 mg/mL	0.01 mg/mL	0.001 mg/mL
Acetone	69.59 ± 2.36	61.26 ± 3.69	59.54 ± 2.85	16.88 ± 1.10
Ethanol	76.36 ± 3.03	67.26 ± 3.18	35.09 ± 2.91	24.13 ± 2.50
Methanol	70.22 ± 3.20	59.74 ± 2.95	42.12 ± 3.44	22.86 ± 2.68
Doxorubicin (10 µM)	78.56 ± 2.87			

## Data Availability

Data will be available on request.

## Sample Availability

Samples of the plant material are available from the authors.

## References

[1] Petrovska B.B. (2012). Historical review of medicinal plants’ usage. Pharmacogn. Rev..

[2] Amiri M.S., Yazdi M.E.T., Rahnama M. (2021). Medicinal plants and phytotherapy in Iran: Glorious history, current status and future prospects. Plant Sci. Today.

[3] Azaizeh H., Fulder S., Khalil K., Said O. (2003). Ethnobotanical knowledge of local Arab practitioners in the Middle Eastern region. Fitoterapia.

[4] Tyler V.E. (1999). Phytomedicines: Back to the Future. J. Nat. Prod..

[5] Pal S.K., Shukla Y. (2003). Herbal medicine: Current status and the future. Asian Pac. J. Cancer Prev..

[6] Khan M.S.A., Ahmad I., Chattopadhyay D., Khan M.S.A., Ahmad I. (2019). Herbal medicine: Current trends and future prospects. New Look to Phytomedicine.

[7] Rizwan K., Khan S.A., Ahmad I., Rasool N., Ibrahim M., Zubair M., Jaafar H.Z., Manea R. (2019). A Comprehensive Review on Chemical and Pharmacological Potential of *Viola betonicifolia*: A Plant with Multiple Benefits. Molecules.

[8] Majeed I., Rizwan K., Ashar A., Rasheed T., Amarowicz R., Kausar H., Zia-Ul-Haq M., Marceanu L.G. (2021). A Comprehensive Review of the Ethnotraditional Uses and Biological and Pharmacological Potential of the Genus *Mimosa*. Int. J. Mol. Sci..

[9] Rizwan K., Majeed I., Bilal M., Rasheed T., Shakeel A., Iqbal S. (2022). Phytochemistry and Diverse Pharmacology of Genus *Mimosa*: A Review. Biomolecules.

[10] Prabhakar K.R., Veeresh V.P., Vipan K., Sudheer M., Priyadarsini K.I., Satish R.B.S.S., Unnikrishnan M.K. (2006). Bioactivity guided fractionation of *Coronopus didymus*: A free radical scavenging perspective. Phytomedicine.

[11] Busnardo T.C.P.M., Padoani C., Mora T.C., Biavatti M.W., Fröde T.S., Bürger C., Claudino V.D., Dalmarco E.M., de Souza M.M. (2010). Anti-inflammatory evaluation of *Coronopus didymus* in the pleurisy and paw oedema models in mice. J. Ethnopharmacol..

[12] Borges M.S., Freitas M.D., Cardoso S., Citadini- V., Bó S.D., Amaral P.D.A. (2021). Ethnobotanical study of selected medicinal plants used for the treatment of respiratory diseases in Southern Brazil. J. Med. Plant Res..

[13] Noreen H., Semmar N., Farman M., McCullagh J.S. (2017). Measurement of total phenolic content and antioxidant activity of aerial parts of medicinal plant *Coronopus didymus*. Asian Pac. J. Trop. Med..

[14] Iqbal D., Javaid A. (2012). Bioassays guided fractionation of *Coronopus didymus* for its antifungal activity against *Sclerotium rolfsii*. Nat. Prod. Res..

[15] Sidhu G.P.S., Bali A.S., Singh H.P., Batish D.R., Kohli R.K. (2020). Insights into the tolerance and phytoremediation potential of *Coronopus didymus* L.(Sm) grown under zinc stress. Chemosphere.

[16] Sidhu G.P.S., Singh H.P., Batish D.R., Kohli R.K. (2016). Effect of lead on oxidative status, antioxidative response and metal accumulation in *Coronopus didymus*. Plant Physiol. Biochem..

[17] Aboulaghras S., Sahib N., Bakrim S., Benali T., Charfi S., Guaouguaou F.-E., Omari N.E., Gallo M., Montesano D., Zengin G. (2022). Health Benefits and Pharmacological Aspects of Chrysoeriol. Pharmaceuticals.

[18] Singh B., Singh S., Singh B., Kitchlu S., Babu V. (2019). Assessing ethnic traditional knowledge, biology and chemistry of *Lepidium didymum* L., lesser-known wild plants of Western Himalaya. Proc. Natl. Acad. Sci. USA India Sec. B. Biol. Sci..

[19] Mumtaz R., Zubair M., Khan M.A., Muzammil S., Siddique M.H. (2022). Extracts of Eucalyptus alba Promote Diabetic Wound Healing by Inhibiting α-Glucosidase and Stimulating Cell Proliferation. Evid.-Based Complement. Altern. Med..

[20] Zubair M., Nybom H., Lindholm C., Rumpunen K. (2011). Major polyphenols in aerial organs of greater plantain (*Plantago major* L.), and effects of drying temperature on polyphenol contents in the leaves. Sci. Hortic..

[21] Hakeem M.L., Bhattacharyya D.N., Campbell I.W. (2010). Diabetes mellitus and travel-related illnesses. Br. J. Diabetes Vasc. Dis..

[22] Idrees R., Fatima S., Abdul-Ghafar J., Raheem A., Ahmad Z. (2018). Cancer prevalence in Pakistan: Meta-analysis of various published studies to determine variation in cancer figures resulting from marked population heterogeneity in different parts of the country. World J. Surg. Oncol..

[23] Bellance N., Lestienne P., Rossignol R. (2009). Mitochondria: From bioenergetics to the metabolic regulation of carcinogenesis. Front. Biosci..

[24] Yoshikawa T., Naito Y. (2002). What is oxidative stress?. Jpn. Med. Assoc. J..

[25] De Souza G.C., Haas A.P.S., Von Poser G.L., Schapoval E.E.S., Elisabetsky E. (2004). Ethnoparmacological studies of antimicrobial remedies in the south of *Brazil*. J. Ethnopharmacol..

[26] Shakoor A., Zaib G., Rahman A. (2018). Biological activities of three medicinal plants from district Mirpur, AJK, Pakistan, *Pak*. J. Pharm. Sci..

[27] Noreen H., Farman M., McCullagh J.S.O. (2016). Bioassay-guided isolation of cytotoxic flavonoids from aerial parts of *Coronopus didymus*. J. Ethnopharmacol..

[28] Das D., Nath B.C., Phukon P., Dolui S.K. (2013). Synthesis of ZnO nanoparticles and evaluation of antioxidant and cytotoxic activity. Colloids Surf. B.

[29] Nair S.S., Kavrekar V., Mishra A. (2013). In Vitro studies on alpha amylase and alpha glucosidase inhibitory activities of selected plant extracts. Eur. J. Exp. Biol..

[30] Khanal P., Patil B. (2019). Gene set enrichment analysis of alpha-glucosidase inhibitors from *Ficus benghalensis*. Asian Pac. J. Trop. Biomed..

[31] Rudrapal M., Khairnar S.J., Khan J., Dukhyil A.B., Ansari M.A., Alomary M.N., Devi R. (2022). Dietary Polyphenols and Their Role in Oxidative Stress-Induced Human Diseases: Insights into Protective Effects, Antioxidant Potentials and Mechanism(s) of Action. Front. Pharmacol..

[32] Akowuah G.A., Ismail Z., Norhayati I., Sadikun A. (2005). The effects of different extraction solvents of varying polarities on polyphenols of *Orthosiphon stamineus* and evaluation of the free radical-scavenging activity. Food Chem..

[33] Chanda S.V., Kaneria M.J. (2012). Optimization of conditions for the extraction of antioxidants from leaves of *Syzygium cumini* L. using different solvents. Food Anal. Methods.

[34] Martins S., Aguilar C.N., Teixeira J.A., Mussatto S.I. (2012). Bioactive compounds (phytoestrogens) recovery from *Larrea tridentata* leaves by solvents extraction. Sep. Purif. Technol..

[35] Anokwuru C.P., Ajibaye O., Adesuyi A.O. (2011). Polyphenolic content and antioxidant activity of *Hibiscus sabdariffa* calyx. Res. J. Medic. Plants.

[36] Spigno G., Tramelli L., De Faveri D.M. (2007). Effects of extraction time, temperature and solvent on concentration and antioxidant activity of grape marc phenolics. J. Food Eng..

[37] Vongsak B., Kongkiatpaiboon S., Jaisamut S., Machana S., Pattarapanich C. (2015). In vitro alpha glucosidase inhibition and free-radical scavenging activity of propolis from Thai stingless bees in mangosteen orchard. Rev. Bras. Farmacogn..

[38] Faden A.A. (2018). Evaluation of antibacterial activities of aqueous and methanolic extracts of *Areca catechu* against some opportunistic oral bacteria. Biosci. Biotechnol. Res. Asia.

